# Decrease of PECAM-1-gene-expression induced by proinflammatory cytokines IFN-γ and IFN-α is reversed by TGF-β in sinusoidal endothelial cells and hepatic mononuclear phagocytes

**DOI:** 10.1186/1472-6793-8-9

**Published:** 2008-05-08

**Authors:** Katrin Neubauer, Alexander Lindhorst, Kyrylo Tron, Giuliano Ramadori, Bernhard Saile

**Affiliations:** 1 University of Göttingen, Department of Internal Medicine, Section of Gastroenterology and Endocrinology, Göttingen, Germany

## Abstract

**Background and aim:**

The mechanisms of transmigration of inflammatory cells through the sinusoids are still poorly understood. This study aims to identify in vitro conditions (cytokine treatment) which may allow a better understanding of the changes in PECAM (platelet endothelial cell adhesion molecule)-1-gene-expression observed in vivo.

**Methods and results:**

In this study we show by immunohistochemistry, that there is an accumulation of ICAM-1 (intercellular cell adhesion molecule-1) and ED1 positive cells in necrotic areas of livers of CCl_4_-treated rats, whereas there are few PECAM-1 positive cells observable. After the administration of CCl_4_, we could detect an early rise of levels of IFN-γ followed by an enhanced TGF-β protein level. As shown by Northern blot analysis and surface protein expression analysed by flow cytometry, IFN-γ-treatment decreased PECAM-1-gene-expression in isolated SECs (sinusoidal endothelial cells) and mononuclear phagocytes (MNPs) in parallel with an increase in ICAM-1-gene-expression in a dose and time dependent manner. In contrast, TGF-β-treatment increased PECAM-1-expression. Additional administration of IFN-γ to CCl_4_-treated rats and observations in IFN-γ^-/- ^mice confirmed the effect of IFN-γ on PECAM-1 and ICAM-1-expression observed in vitro and increased the number of ED1-expressing cells 12 h after administration of the toxin.

**Conclusion:**

The early decrease of PECAM-1-expression and the parallel increase of ICAM-1-expression following CCl_4_-treatment is induced by elevated levels of IFN-γ in livers and may facilitate adhesion and transmigration of inflammatory cells. The up-regulation of PECAM-1-expression in SECs and MNPs after TGF-β-treatment suggests the involvement of PECAM-1 during the recovery after liver damage.

## Background

Inflammatory cells leave the circulation at sites of local inflammation through a series of distinct sequential steps that are classified as rolling, activation, tight adhesion, transmigration and passage across the basement membrane [1-6]. Due to the specialized sinusoids, inflammatory processes in the liver are thought to differ from those in other organs. It has been suggested that SECs of the normal liver – in contrast to capillary endothelial cells – only express a restricted set of adhesion molecules [7-9].

PECAM-1 is known to be expressed on the surface of endothelial cells, circulating platelets, monocytes, granulocytes and some subsets of T-lymphocytes. PECAM-1 has been proposed as one of the main players in transendothelial migration of neutrophils, monocytes, and natural killer cells in both in vivo and in vitro models, since by the use of antibodies directed against PECAM-1, transmigration and inflammation could be significantly reduced [10-12]. However, the pathomechanisms involving PECAM-1 are still a matter of debate, since in a PECAM-1-knock-out-mouse model, transmigration of inflammatory cells and inflammatory damage were almost unchanged [13].

In our previous studies, we have demonstrated that PECAM-1 is expressed in SECs and MNPs of the rat liver. Furthermore, we have shown that the PECAM-1 expression in SECs and MNPs is decreased after CCl_4_-treatment in vivo before inflammatory phagocytes accumulate in the pericentral area of rat livers and following IFN-α-treatment of cultured SECs and MNPs in vitro. In contrast, ICAM-1-gene expression is upregulated early after CCl_4 _administration, reaching a maximum after 24 h. Thereafter, ICAM-1 is decreasingly expressed in SECs, reaching the control level of the value at 0 h after 48–72 h. In MNPs ICAM-1 is maximally expressed 6 h after CCl_4 _administration and thereafter expression declines to control levels 72 h after CCl_4 _administration. [14,15].

Since we have seen that there is a down-regulation of PECAM-1 in the rat liver after the administration of CCl_4_, in this study we aimed to identify further cytokines that are responsible for the down-regulation of PECAM-1 on SECs and on transmigrating mononuclear phagocytes (MNPs), but also to identify cytokines that allow the reversal of this effect on both cell types.

A pivotal role in pathomechanisms of liver injury has been assigned to IFN-γ next to IFN-α [16] and it has been demonstrated that IFN-γ can induce IFN-α expression in MNPs [17]. TGF-β is a known pleiotropic cytokine with profibrogenic and anti-inflammatory capacities [18]. In this study we measured the concentration of IFN-γ and TGF-β in the rat liver at different time points after CCl_4_-administration paying particular attention to the early time points. Furthermore we tested the modulation of PECAM-1 and ICAM-1-expression in SECs and MNPs following IFN-γ-treatment in vitro and in vivo. We also studied the effect of TGF-β on PECAM-1 expression in isolated SECs and MNPs. Whereas IFN-γ reduces PECAM-1 expression in parallel with the increase of ICAM-1 expression, TGF-β up-regulates both molecules and is also able to counteract the down-regulating effect of IFN-γ on PECAM-1 expression in vitro.

These data strongly suggest an early down-regulation of PECAM-1 in parallel to an ICAM-1 up-regulation as a possible important mechanism for the adhesion and transmigration of inflammatory cells. However, further in vitro and in vivo studies are required to confirm this hypothesis. The TGF-β induced PECAM-1 up-regulation may be of importance during the recovery phase.

## Results

### PECAM-1-immunoreactivity in livers from normal rats and from rats treated with CCl_4_: Comparison to ED1-, and ICAM-1-immunoreactivity

**Figure 1:**
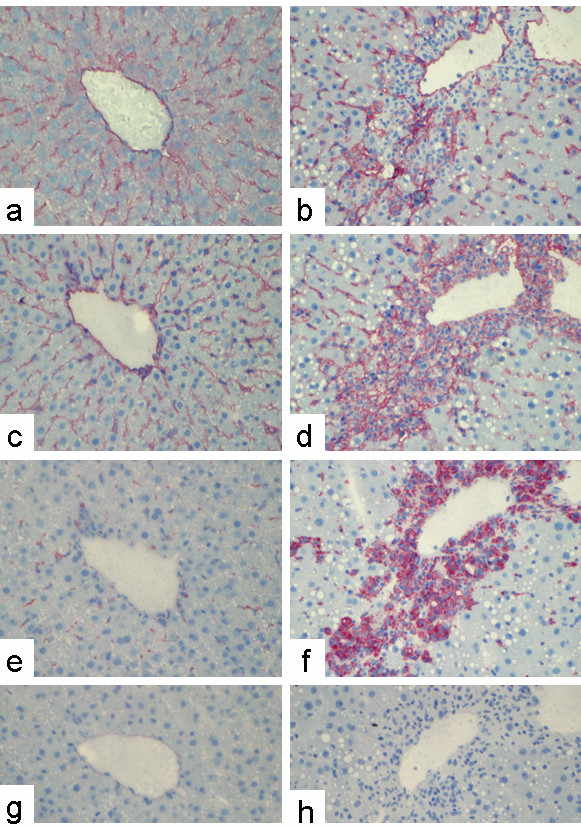
**Indirect immunodetection of PECAM-1, ICAM-1, and ED1 in sections of normal rat or acutely damaged livers (48 h after a single CCl_4_-administration).** Sections of normal rat liver tissue (a, c, e) or acutely damaged rat liver tissue (b, d, f) were stained with a monoclonal antibody directed against rat PECAM-1 (a, b), ICAM-1 (c, d), ED1 (e, f), followed by the APAAP immunodetection (Original magnification × 250). Figures 1g and 1h are the negative control, which was performed with murine serum. Note that in the necrotic areas around the central vein most of the ED1-positive cells representing inflammatory MNPs are PECAM-1 negative, but ICAM-1 positive.

Immunostaining of sections from a normal rat liver using a monoclonal antibody against rat PECAM-1 revealed positivity along the sinusoids in a similar pattern to ICAM-1 (Figure 1), suggesting PECAM-1 positivity of SECs. Staining of sequential sections by antibodies against PECAM-1 and against markers of MNPs (ED1) suggests that the positivity along the sinusoids of the normal rat liver is also partly due to MNPs (Figure 1).

To analyze PECAM-1 immunoreactivity under conditions of inflammation, we used a model of CCl_4_-induced liver damage, which is characterized by the accumulation of inflammatory cells (mostly MNPs) starting 12 h after the administration of CCl_4 _and peaking after 48 h, when pericentral necrotic areas can be seen. The possibility that some PECAM-1 positive cells are myofibroblasts (MFB), activated HSCs (hepatic stellate cells), or liver myofibroblasts deriving from cells different from quiescent HSCs can be excluded since isolation of these cell types at different time points after CCl_4 _administration and primary cultures of HSC or liver myofibroblasts from normal rat livers never showed any PECAM-1-gene expression (data not shown).

At 48 h after a single dose of CCl_4 _there were significant numbers of ICAM-1 and ED1 positive cells within the necrotic pericentral areas (Figure 1). However, the ICAM-1- and ED1-immunoreactive cells of the necrotic area were PECAM-1 negative (Figure 1).

### Expression of IFN-γ, IFN-α and TGF-β in CCl_4_-treated rat livers

**Figure 2:**
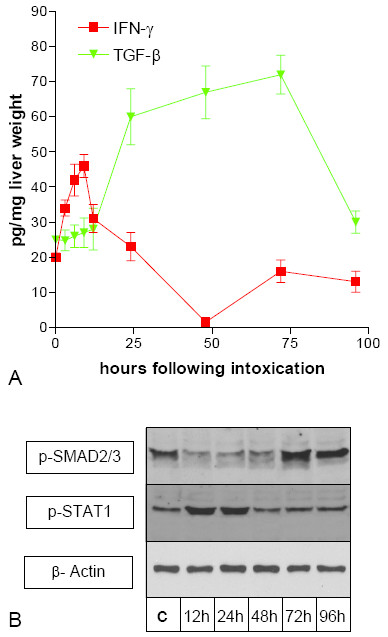
**(A) Expression of TNF-α, IFN-γ, and TNF-β during acute liver damage (CCl_4_-model). **Livers taken at different time points after a single administration of CCl_4 _were homogenized and used with a human-TNF-α and human-TNF-β EIA-kit or rat-IFN-γ ELISA-kit. Cytokine concentration is indicated as pg per mg of liver wet weight. Error bars indicate standard deviation of the mean value of four experiments from four different animals. (B) Western Blot analysis of p-SMAD2/3 and p-STAT-1 in SECs during acute liver damage (CCl_4_-model). SECs from untreated control rats (c) and different time points after CCl_4 _administration were isolated. β-actin was used as loading control (not shown). Consistent data could be shown in SECs isolated from two different rats for each time point.

During recent years, an important role for IFN-γ and TGF-β in acute and chronic liver damage has been suggested (for review see [19-21]). Since we aimed to identify cytokines responsible for the modulation of PECAM-1 and ICAM-1 in vivo, we measured the concentrations of IFN-γ and TGF-β protein in liver homogenates after a single CCl_4_-administration using EIA or Elisa. Compared to the livers of untreated controls (time point 0 h), there was an early steep increment in IFN-γ expression with a maximum 9 h after CCl_4 _administration, reaching a level of 46 pg per mg of liver wet weight. At 24 h after CCl_4 _administration the IFN-γ concentration peaked at the level of the control livers. At 96 h after CCl_4 _administration the IFN-γ expression returned to the level seen in the livers of untreated rats. (Figure 2). TGF-β expression only slightly increased during the first 12 h after CCl_4_-administration. However it increased significantly thereafter with a maximum after 72 h, reaching a level of 67 pg per mg of liver wet weight and returned to the level seen in the livers of untreated rats after 96 h (Figure 2).

Since measurement of cytokine levels in over the whole liver gave no information on possible local variations in concentration we performed Western Blot analysis of p-STAT1 and p-SMAD2/3 in SECs isolated at different time points after CCl_4 _administration. p-STAT1 was found to be increased at 12 h and 24 h after CCl_4_^-^administration, indicating that a receptor-mediated IFN signal transduction lasts longer than suspected from the cytokin levels of the total liver. In order to investigate at which time points after CCl_4_-administration TGF-β induces intracellular signaling in SECs we performed Western Blot analysis of p-SMAD2/3. Whereas TGF-β expression is increased between 24 h and 72 h after CCl_4_-administration, p-SMAD2/3 is decreased in SECs isolated 24 h–48 h after CCl_4_-administration. Thereafter p-SMAD2/3 is found to be increased.

### IFN-γ decreases PECAM-1 transcript level in liver SECs and MNPs

**Figure 3:**
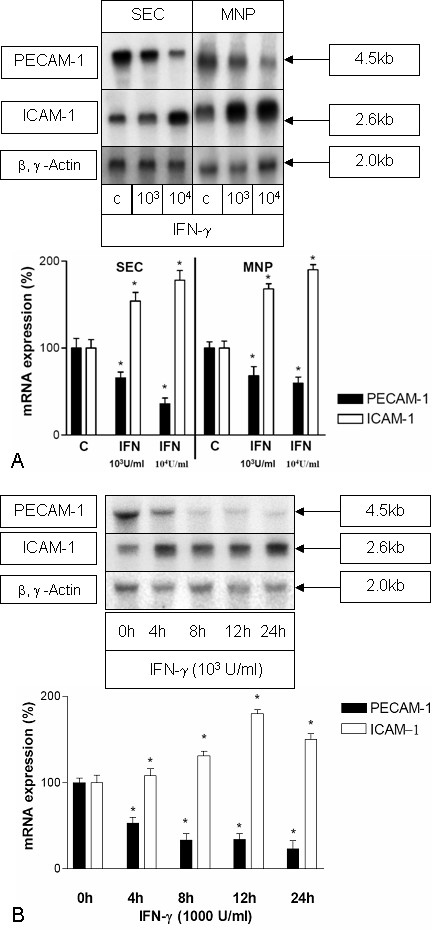
**(A) IFN-γ decreases PECAM-1 transcript level and elevates the ICAM-1 transcript level in liver SECs or MNPs.** Northern blot analysis of total RNA extracted from primary cultures of SECs or MNPs at 24 h after isolation either untreated (0 h) or treated with IFN-γ (1000 (10^3^) or 10000 (10^4^) U/ml) for 12 h. Five μg of total RNA was separated on agarose gel, blotted and hybridised with a ^32^P-dCTP-labeled cDNA probe specific for rat PECAM-1. Same membranes were hybridised with a cDNA specific for rat ICAM-1 or for β- and γ-actin. This figure shows results of five experiments from five different isolations. The lower panel shows a statistical evaluation of densitometric scans. Error bars indicate standard deviation of the mean value after normalization on β,γ-actin expression. The P-value was tested to show significant differences of control cells compared to cells treated with IFN-γ. * indicates p < 0.05. (B) Northern blot analysis of total RNA extracted from primary cultures of SECs at day 1 after isolation treated with IFN-γ (1000 U/ml) for 4 h, 8 h, 12 h or 24 h. Five μg of total RNA was separated on agarose gel, blotted and hybridised with a ^32^P-dCTP-labeled cDNA probe specific for rat PECAM-1. The same membranes were hybridised with a cDNA specific for rat ICAM-1 or for β- and γ-actin. (c) The untreated control. The lower panel shows a statistical evaluation of densitometric scans. Error bars indicate standard deviation of the mean value normalized on β,γ-actin expression of five experiments from five different isolations of each cell type. P-value was tested to show significant differences of control cells compared to cells treated with IFN-γ. * indicates p < 0.05.

We analysed the modulation of the PECAM-1 specific transcript level in cultured SECs and MNPs following treatment with IFN-γ (Figure 3). SECs at day one after isolation were cultured in the absence or presence of IFN-γ under serum reduced conditions (0.3% FCS). Testing different concentrations of IFN-γ (100, 1000, 10000 U/ml) indicated that the strongest effects of IFN-γ on the PECAM-1 transcript level were found at a concentration of 10000 U/ml. As shown in Figure 3A, IFN-γ causes a decrease in the PECAM-1 specific transcript level. In contrast the ICAM-1 mRNA level in SECs was increased by IFN-γ-treatment. Furthermore, different stimulation periods (4 h, 8 h, 12 h, and 24 h of incubation with IFN-γ) were compared indicating maximal effects on PECAM specific transcripts after 8 to 12 h of stimulation. It is noteworthy, that an increase of ICAM-1 and a parallel decrease of PECAM-1 could already be observed after 4 h of treatment (Figure 3B) suggesting that the regulation of the two genes may take place at the same time.

Since we were interested in the modulation of PECAM-1 by IFN-γ not only on SECs but also on the transmigrating MNPs, we also tested MNPs. In MNPs from normal rat livers at day 1 after isolation cultured in the absence or presence of IFN-γ (100, 1000, 10000 U/ml) a maximal decrease of PECAM-1 specific transcripts was revealed using 10000 U/ml of IFN-γ for 12 h. At the same time the ICAM-1 mRNA level was increased in MNPs following IFN-γ-treatment (Figure 3A).

### TGF-β-treatment increases PECAM-1 transcript level in cultured SECs and MNPs from normal rat livers

**Figure 4:**
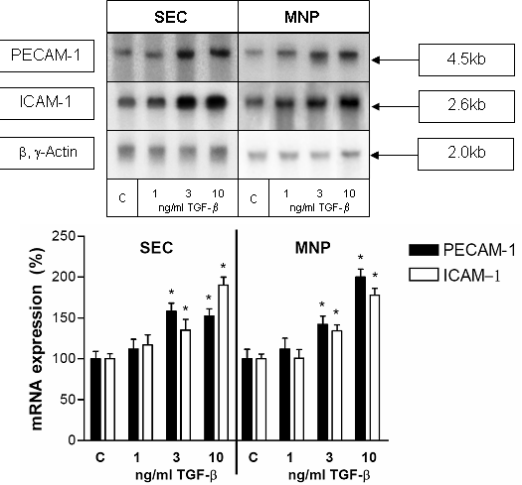
**TNF-β increases the PECAM-1 transcript level and ICAM-1 transcript level in liver SECs and MNPs. **Northern blot analysis of total RNA extracted from primary cultures of SECs or MNPs at day 1 after isolation either untreated (c) or treated with TNF-β for 24 h (in a concentration of 1, 3 and 10 ng/ml). Five μg of total RNA was separated on agarose gel, blotted and hybridised with a ^32^P-dCTP-labeled cDNA probe specific for rat PECAM-1. The same membranes were hybridised with a cDNA specific for rat ICAM-1 or for β- and γ-actin. This figure shows results for five experiments from five different isolations. The lower panel shows a statistical evaluation of densitometric scans. Error bars indicate standard deviation of the mean value normalized on β,γ-actin expression of five experiments from five different isolations of each cell type. P-value was tested to show significant differences of control cells compared to cells treated with TNF-β. * indicates p < 0.05.

Since we had noted the TGF-β levels increasing during the recovery phase of CCl_4 _induced liver injury, we wanted to analyze whether TGF-β could have reverse effects on PECAM-1-expression compared to IFN-γ. When SECs or MNPs were cultured in the presence of TGF-β for different time periods (4 h, 8 h, 12 h, and 24 h) a maximal increase of PECAM-1 specific transcript level was reached after 12 h to 24 h of stimulation. In addition, cells were treated with TGF-β in different concentrations (1, 3 and 10 ng/ml) for 12 hours under serum reduced conditions (0.3% FCS). While no effect of TGF-β on the PECAM-1 mRNA-level was detectable at a concentration of 1 ng/ml, treatment with TGF-β at a concentration of 3 or 10 ng/ml resulted in an increased PECAM-1 specific transcript level in SECs and in MNPs (Figure 4). The strongest effects were revealed using 10 ng/ml of TGF-β. An increase of ICAM-1 specific transcript levels was observed in SECs following the treatment with TGF-β. There was no significant increase in ICAM-1 specific transcript level in MNPs.

**Figure 5:**
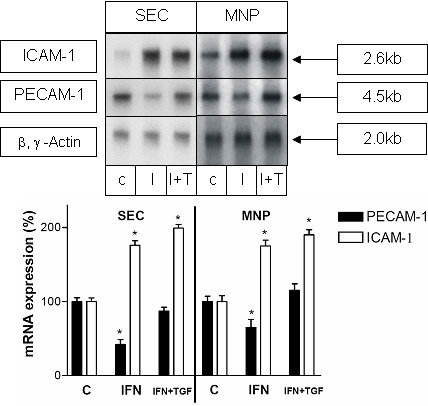
**TNF-β reverses the effect of IFN-γ on PECAM-1 transcript level but has no additional effect on the ICAM-1 transcript level in liver SECs and MNPs.** Northern blot analysis of total RNA extracted from primary cultures of SECs and MNPs at day 1 after isolation either untreated (c) or treated with IFN-γ 10000 (10^4^)U/ml (I) or IFN-γ together with TNF-β (10 ng/ml) (I+T) for 12 h. Five μg of total RNA was separated on agarose gel, blotted and hybridised with a ^32^P-dCTP-labeled cDNA probe specific for rat PECAM-1. The same membranes were hybridised with a cDNA specific for rat ICAM-1 or for β- and γ-actin. This figure shows results of five experiments from five different isolations. The lower panel shows a statistical evaluation of densitometric scans. Error bars indicate standard deviation of the mean value after normalization on β,γ-actin expression. P-value was tested to show significant differences of control cells compared to cells treated with IFN-γ or IFN-γ +TNF-β. * indicates p < 0.05.

In order to investigate whether TGF-β is capable of reversing the effect of IFN-γ on PECAM-1 and ICAM-1 expression on SECs or MNPs or whether both cytokines simply have inverse but independent effects we additionally cultured SECs or MNPs with IFN-γ and TGF-β. Northern Blot analysis revealed that coadministration of IFN-γ and TGF-β inhibits the PECAM-1-downregulating effect of IFN-γ on both cell types. On the other hand coadministration of both cytokines had no additional effect on upregulation of ICAM-1 (Figure 5).

### Surface expression of PECAM-1 and ICAM-1 on SECs and MNPs following IFN-α-, IFN-γ – or TGF-β-treatment

To demonstrate that IFN-γ – or TGF-β treatment also influences PECAM-1 and ICAM-1 surface expression, we further investigated the PECAM-1-gene-expression in SECs and MNPs following treatment with IFN-γ or TGF-β by flow cytometry.

**Figure 6:**
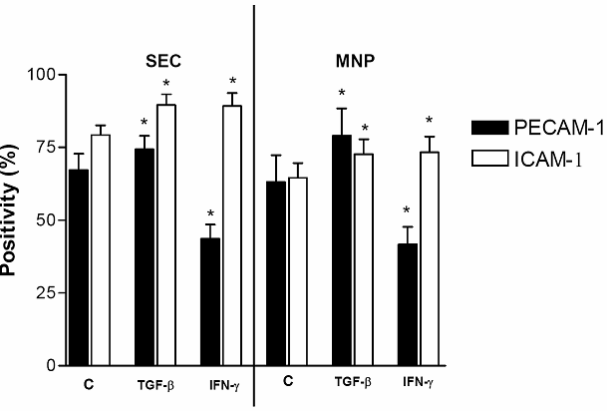
Surface expression of PECAM-1 and ICAM-1 in SECs and MNPs under the treatment of TNF-β or IFN-γ Surface expression of PECAM-1 and ICAM-1 in SECs.isolated from control rats taken for 1 day in culture under the treatment of TNF-β 10(ng/ml) or IFN-γ 1000 U/ml) for the last 12 h. Error bars indicate standard deviation of the(mean value (n = 4). P-value was tested to show significant differences of control cells compared to cells treated with IFN-γ or TNF-β. * indicates p < 0.05.

SECs and MNPs were isolated from rat livers and taken in culture for 24 h, cultured in the presence or absence of IFN-γ or TGF-β for 12 h and processed for flow cytometry. In accordance with the data from the Northern blot analysis, IFN-γ treatment reduced the PECAM-1-expression in both SECs and MNPs (Figure 6) and at the same time induced the expression of ICAM-1, whereas TGF-β-treatment increased PECAM-1- and ICAM-1-expression on SECs. On MNPs only an insignificant increase in ICAM-1 expression was observed.

### Constant PECAM-1 specific transcript level in CCl_4_-treated IFN-γ^-/- ^mice livers and reduced increase of the ICAM-1 specific transcript level

**Figure 7:**
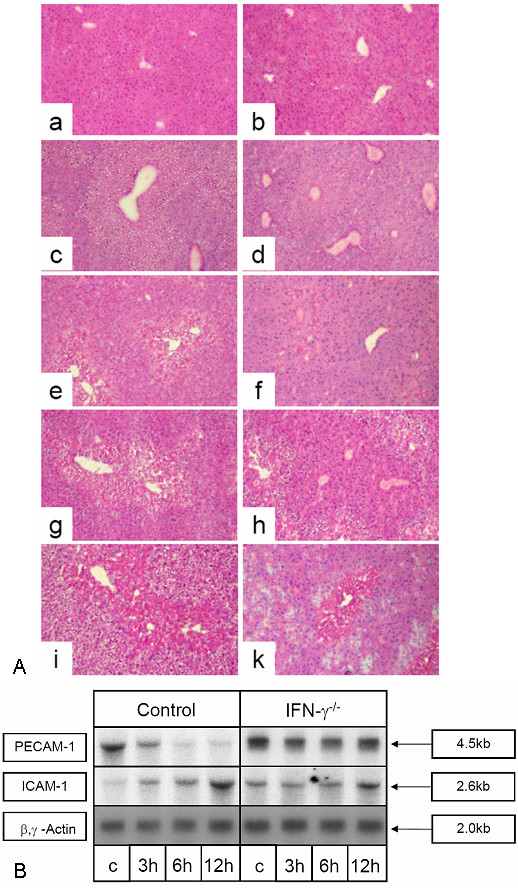
**(A) HE staining of liver sections of C57BL/6 mice (a, c, e, g, i) and B6.**129S7-Ifngtm1Ts (IFN-γ^-/-^) mice (b, d, f, h, k). Sections of normal livers (a, b) or acutely damaged livers (after a single CCl_4_-administration) at time points 12 h (c, d), 24 h (e, f), 48 h (g, h) and 72 h (i, k) are given. (B) Lack of decrease of PECAM-1 specific transcript level in CCl_4_-treated IFN-γ^-/- ^mice livers and reduced increase of the ICAM-1 specific transcript level. C57BL/6 mice (controls) and B6.129S7-Ifngtm1Ts (IFN-γ^-/-^) mice were treated with CCl_4 _and livers were taken for analysis 3 h (3 h), 6 h (6 h) or 12 h (12 h) after treatment (each time point two animals). (c) The untreated control. 10 μg of total RNA was separated on agarose gel, blotted and hybridised with a ^32^P-labeled cDNA probe specific for mouse PECAM-1. β,γ-actin was used as loading control. This figure shows results for 2 experiments of two animals sacrificed at each time point.

For a functional proof that IFN-γ leads to an early decrease of PECAM-1 and to an early increase of ICAM-1 specific transcripts in vivo we used an IFN-γ^-/- ^mice knockout model (B6.129S7-Ifngtm1Ts) [22]. In control mice (female C57BL mice), similar CCl_4_-administration levels to those previously used in rats leads to an early decrease of PECAM-1 specific transcripts and to an increase of ICAM-1 specific transcripts. Whereas an increase of ICAM-1 specific transcripts can also be observed in IFN-γ^-/- ^mice livers after CCl_4_-administration, no significant decrease of PECAM-1 specific transcripts was evident (Figure 7A). A histological comparison after inducing acute liver damage by CCl_4 _showed that the degree of damage in the IFNγ^-/- ^mice was less than in the control mice. Furthermore the damaged pericentral areas were smaller, less fatty and the occurrence of necrotic areas, which were also smaller in the liver sections from IFNγ^-/- ^mice, was observed later (48 h vs. 24 h after CCl_4 _administration). Even 72 h after CCl_4 _administration the hepatocytes are more vital (Fig. 7B).

### Treatment of rats with IFN-γ enhances the decrease of PECAM-1-gene-expression in CCl_4_-treated rat livers in parallel to an enhanced increase of the ICAM-1 gene-expression

**Figure 8:**
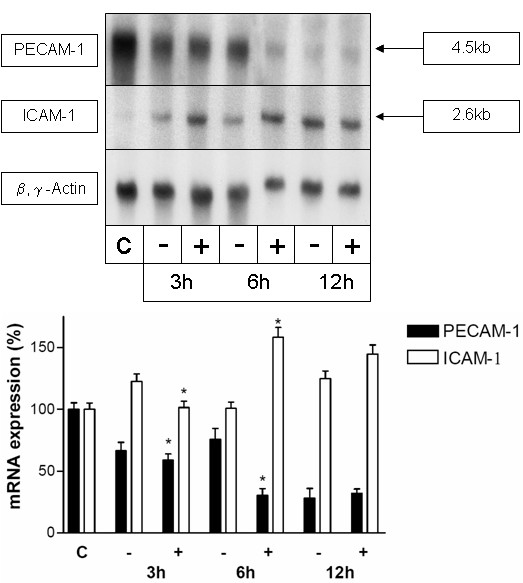
**Treatment of rats with IFN-γ enhances the decrease of the PECAM-1 specific transcript level in CCl_4_-treated rat livers in parallel to an enhanced increase of the ICAM-1 specific transcript level.** Animals were treated with CCl_4 _(-) or with CCl_4 _and IFN-γ (+) and livers were taken for analysis 3 h (3 h), 6 h (6 h) or 12 h (12 h) after treatment (each time four animals). (c) The untreated control. 10 μg of total RNA was separated on agarose gel, blotted and hybridised with a ^32^P-labeled cDNA probe specific for rat PECAM-1. The same membranes were hybridised with a cDNA specific for rat ICAM-1 or for β- and γ-actin. This figure shows the results for four experiments. The lower panel shows a statistical evaluation of densitometric scans. Error bars indicate standard deviation of the mean value normalized on β,γ-actin expression of five experiments from five different isolations of each cell type. P-value was tested to show significant differences of rats 12 h after treatment with CCl_4 _compared to treatment with CCl_4 _and IFN-γ. * indicates p < 0.05.

To further analyse whether the effects of IFN-γ observed in vitro in the previous experiments can be reproduced in vivo, rats were treated either with CCl_4 _or with IFN-γ and CCl_4 _in parallel. Measurement of the IFN-γ concentration in liver homogenates by means of ELISA revealed an increase of 5–10% in the IFN-γ concentration in liver tissue following the intraperitoneal treatment with 50000 U IFN-γ. Since, from the in vitro and in vivo experiments we expected the effects of IFN-γ within the first 10 h after treatment, livers were analyzed (3 h, 6 h, 12 h after treatment) for changes in PECAM-1 and ICAM-1-gene-expression by Northern blot analysis (Figure 8).

When rats were treated with IFN-γ and CCl_4 _the increase of ICAM-1 transcript level and the decrease of PECAM-1 transcript level was enhanced compared to rats treated with CCl_4 _alone. This difference between rats treated with CCl_4 _and IFN-γ to rats treated with CCl_4 _alone was only detectable up to 6 h following the treatment.

### Treatment of rats with IFN-γ enhances the accumulation of ED1-expressing cells in CCl_4_-treated rat livers

**Figure 9:**
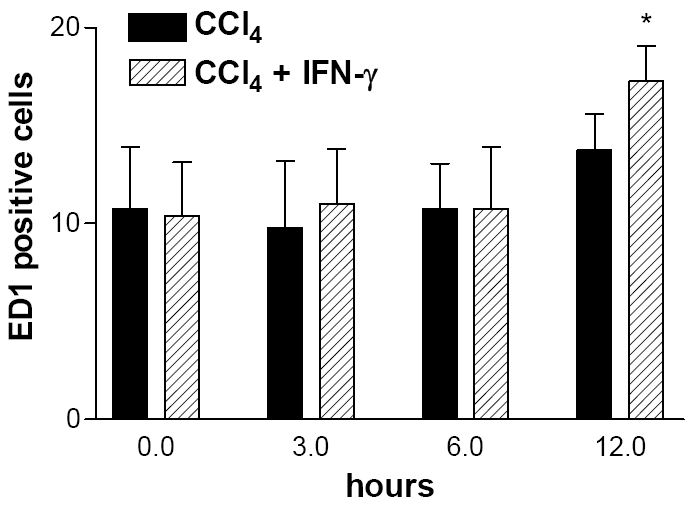
**Treatment of rats with IFN-γ enhances the number of ED1 positive cells. **Animals were treated with CCl_4 _or with CCl_4 _and IFN-γ and livers were taken for analysis 3 h (3 h), 6 h (6 h) or 12 h (12 h) after treatment. Sections were stained by indirect immunohistochemistry using a monoclonal antibody against ED1. Positive cells were counted in 10 purviews (250 × magnification). Error bars indicate standard deviation of the mean value of 10 counted purviews. P-value was tested to show significance differences of rats 12 h after treatment with CCl_4 _compared to treatment with CCl_4 _and IFN-γ. * indicates p < 0.05.

Since differences in adhesion molecule expression following the additional treatment with IFN-γ could be observed up to 6 h after treatment, sections of rat livers were analysed by indirect immunostaining with a monoclonal antibody against the ED1 antigen at 3 h, 6 h, and 12 h after the treatment to analyze whether the amount of recruited inflammatory cells was influenced. The staining of control livers revealed the previously observed pattern of non-parenchymal cells situated around the vessels. At 12 h after CCl_4_-treatment the number of ED1-positive cells around the vessels and along the sinusoids increased. Interestingly, when rats were treated with the combination of IFN-γ and CCl_4 _the number of ED1 positive cells at 12 h was significantly higher than in the livers of animals treated with CCl_4 _alone (Figure 9).

## Discussion

Since we have seen that there is a down-regulation of PECAM-1 in rat livers after the administration of CCl_4_, in this study we aimed to identify cytokines that were responsible for the down-regulation of PECAM-1 on SECs and on transmigrating MNPs, but we also wanted to identify cytokines that allow the reversal of this effect on both cell types. In this study, we show that in CCl_4_-treated rat livers there is an early rise of levels of IFN-γ and – during the recovery phase – an increase of TGF-β-protein level. Similar observations for TGF-β levels after induced liver damage have also been reported by others [23,24]. However especially measurements of the early time points are missing in the literature. This might be the reason for reports of a decrease of IFN-γ after inducing liver damage [25]. In vitro studies indicated that IFN-γ-treatment induced a decrease of PECAM-1-gene-expression in parallel with an increase in ICAM-1-gene-expression. Furthermore, TGF-β-treatment resulted in an increase of PECAM-1-expression in SECs and MNPs. TGF-β – also inhibited the effect of IFN-γ treatment. However, the temporal course of the cytokine expression in vivo after administration of the damaging noxae does not perfectly match the temporal course of changes in the expression of PECAM-1 or ICAM-1. This has two major causes. Firstly, in vivo, many intercellular contacts and additional cytokines have an impact on PECAM-1 and ICAM-1 expression on SECs and MNPs e.g. IFN-α [15] which shows a similar expression profile compared to IFN-γ. Secondly, local differences in the concentration of the cytokines can lead to increased levels of the cytokines around SECs or MNPs when levels in the total liver are already decreased. The observation that IFN-γ or TGF-β signalling as measured by p-STAT1 or p-SMAD2/3 is delayed and can be observed when the levels of IFN-γ or TGF-β are already decreasing supports this thesis.

The in vitro effect of IFN-γ could be confirmed in vivo, since the administration of IFN-γ (0,4 μg per mg liver wet weight, which is comparable to the concentration used for the in vitro treatment (0.01 to 0.1 μg/ml of IFN-γ)), together with CCl_4 _further decreased the PECAM-1 transcript level and also further increased the number of ED1-positive cells in the liver at 12 h after the administration of CCl_4 _compared to the single CCl_4 _administration to the animals. In contrast, in IFN-γ^-/- ^mice the lack of IFN-γ may be responsible for the missing early decrease of PECAM-1 and the reduced increase of ICAM-1.

To study the involvement of cell adhesion molecules during inflammation and tissue damage in vivo several approaches have been used. It has been difficult to block inflammation using reagents directed against single molecules. Several studies, however, revealed that the transmigration of monocytes and of neutrophils could be inhibited using antibodies against PECAM-1 in vitro [12], and in vivo, as the administration of antibodies against PECAM-1 or the administration of the soluble domain 1 of PECAM-1 blocked the accumulation of leukocytes in different models of inflammation [10,11,26,27]. In PECAM-1-knock-out-mice, however, only a slowing of inflammatory cells transmigrating through the basement membrane was observed. This slowing had no effect on the total number of transmigrated cells [13].

In vivo data showing a reduced PECAM-1 expression in inflammatory cells have been interpreted as if PECAM-1 down-regulation were a consequence of the transmigration [28]. Our results demonstrate that in vitro and in vivo treatment with IFN-γ down-regulates the expression of PECAM-1 in liver cells at an early time point. The in vitro studies reveal a decreased PECAM-1-expression on SECs and on MNPs. These data support the hypothesis that PECAM-1 has to be down-regulated to facilitate transmigration. A confirmation of this hypothesis could be seen in a model of abdominal aortic transplantation. The transmigration of MNPs was unexpectedly enhanced when aortic allografts from PECAM-1 knock-out-mice were transplanted compared to transplanted allografts from wild type mice [29]. The authors suggested that this phenomenon might be due to the fact that PECAM-1 is concentrated at interendothelial junctions and is thereby important for the structural stability of the endothelial barrier. In PECAM-1 deficient grafts this endothelial stability could be reduced during the inflammatory episode after transplantation making it easier for macrophages to transmigrate through the endothelium.

Another relevant finding of the in vivo experiments is the increase of TGF-β concentration in the liver during the recovery phase after CCl_4_-administration, when IFN-γ – and IFN-α concentration have already been decreased. TGF-β is a pleiotropic cytokine with profibrogenic and anti-inflammatory capacities [18]. The data presented in this study show that in cultured SECs and MNPs, TGF-β increases PECAM-1-expression. Furthermore, TGF-β inhibited the effect of IFN-γ on the PECAM-1 transcript level. Although an up-regulation of PECAM-1-gene-expression by TGF-β-treatment has been observed using a human macrophage cell line (U937 cells) [30], this is the first report showing similar effects on endothelial cells and on tissue macrophages. These data indicate a different modulation of PECAM-1 by IFN-γ and TGF-β in SECs and MNPs. Under normal conditions SECs are an efficient barrier against corpusculate matter. Early production of IFN-γ after administration of toxic agents could not only induce an increase of ICAM-1 expression, but also a decreased PECAM-1 expression, which is essential for the tight adhesion and for the transmigration of inflammatory cells into the parenchyma. During liver repair cytokines such as TGF-β may be effective in reestablishing the endothelial barrier.

Whereas IFN-γ has previously been shown to exert antifibrotic effects in man and mice by abrogating TGF-β signalling [31,32], inducing an acute liver damage in IFN-γ knockout mice revealed that the degree of damage was reduced and delayed when compared to control mice. This suggests that, least in the early time points after administration of a damaging noxae, IFN-γ is involved in an increase of liver damage without antagonizing TGF-β. From these data, blockage of IFN-γ signalling in the early time points after liver damage, thereby inhibiting downregulation of PECAM, could be a valuable tool in the treatment of patients during the early phases of liver damage.

## Methods

### Animals

Male Wistar rats (8 weeks old) (Charles River, Sulzberg, Germany), female C57BL/6 mice and B6.129S7-Ifngtm1Ts (IFN-γ-deficient) mice (The Jackson Laboratory, Bar Harbor, ME) received humane care and were kept according to the institution's and the National Institutes' of Health guidelines.

### Reagents

Human recombinant IFN-γ was purchased from R&D systems (Wiesbaden, FRG). Human TGF-β and IFN-α were purchased from Sigma (Deistendorfen, FRG). The monoclonal antibody against rat PECAM-1 was purchased from Natutec (Frankfurt, FRG). The monoclonal antibodies against rat ICAM-1 was from Genzyme (Cambridge, MA, USA), against pSTAT1, pSMAD2/3 and β-actin were from Santa Cruz Biotechnology (Heidelberg, Germany) and against ED1 was from Biermann (Biermann, Wiesbaden, Germany).

### cDNA probes

Rat and mouse PECAM-1 and ICAM-1 specific cDNAs were generated as described earlier [14,15]. Northern blot results were normalised to an oligonucleotide probe specific for 28S rRNA [33] or with a chicken α-actin cDNA exhibiting cross reactivity with β- and γ-actin, which was a gift from A. Schwartz [34]. Furthermore, a PCR-generated cDNA directed against rat IFN-α was used mapping positions 140–509 of the published sequence [35].

### Inducing liver damage

Acute liver damage was induced in 8 weeks old male Wistar rats (body weight about 200 g) by oral administration of a carbon-tetrachloride/maize oil solution (50% v/v) as described [36-38]. Control animals were treated with maize-oil only. Six animals in each group were sacrificed 3 h, 6 h, 9 h, 12 h, 24 h, 48 h, 72 h, and 96 h after a single high dose CCl_4 _administration. Furthermore, 16 animals were treated with IFN-γ (50000 IU/100 g body weight which is about 0.4 μg per mg of rat liver, assuming that about 80% of the intraperitoneally injected IFN-γ reaches the liver) in addition to CCl_4_-treatment. Animals were sacrificed 3 h, 6 h, 12 h, and 24 h after the treatment, since we expected IFN-γ induced modulation of gene expression to take place at early time points after the administration. For inducing liver damage in mice a carbon-tetrachloride/maize oil solution (50% v/v) was administrated intraperitoneally with a concentration of 1 ml CCl_4_/kg.

### Immunohistology

APAAP-immunostaining was performed as described [39]. Negative controls were performed by replacing the primary antibody by murine IgGs. ED1 positive cells were counted in 10 purviews (250 × magnification) of immunostainings of two independent series of experiments.

### Measurement of IFN-γ, IFN-α and TGF-β in liver tissue

Liver specimens (40 mg) were homogenized and used with a human-IFN-α and-TGF-β EIA-kit (Chemicon, Tamemcula, USA) or rat-IFN-γ ELISA-kit (Genzyme Diagnostics, Cambridge, Massachusetts, USA). Measurements were carried out according to the manufacturers' protocols.

### Cell isolation and culture conditions

MNPs from normal rat livers were isolated according to the method of De Leeuw et al. [40] as described previously [41]. SECs from normal rat livers were obtained according to Knook et al. [42] with modifications as described [14,15,36]. MNPs were cultured in M-199 supplemented with 10% FCS. SECs were cultured on collagen-coated tissue culture plates in endothelial cell medium (Promo cell, Heidelberg, FRG) supplemented with epidermal growth factor (10^-11 ^mg/ml), basic fibroblast growth factor (10^-9 ^mg/ml), 0.15% insulin and 2% FCS. The medium was replaced every day. Purity of SECs was more than 91% as tested by incorporation of dioctadecyl tetramethyl indocarbocyanine perchlorate acetylated low density lipoprotein and electron microscopy of SECs as described in [14,15,43]. Freshly isolated MNPs from normal livers were more than 99% viable and more than 98% pure (as tested by ED1/ED2 staining). On day 1 of culture, SECs and MNPs were washed three times with Gey's balanced salt solution and were incubated in serum reduced (0.3% FCS) culture medium with increasing concentrations of IFN-α (10, 100, 1000, U/ml), IFN-γ (10, 100, 1000, 10000 U/ml which is 0.01 to 0.1 μg/ml), or TGF-β (1, 3, and 10 ng/ml).

### Flow cytometric quantification of PECAM-1 and ICAM-1 expression in isolated liver cells

For quantification of PECAM-1 and ICAM-1 immunofluorescence after indirect immunostaining, we used flow cytometry of MNPs and SECs on day one after isolation (Epics ML, Coulter, Kerfeld, Germany). Negative controls were performed using murine IgGs instead of the primary antibody.

### Northern Blot analysis of total RNA

Total RNA was isolated according to Chirgwin [44] and was separated by agarose gel electrophoresis, transferred onto nylon membranes and hybridised with specific ^32^P dCTP or ^32^P dATP labeled cDNA probes as described [43]. cDNA probes were labelled by Random priming^®^. The oligo recognizing the 28S rRNA was end labelled using ^32^P dATP. Hybridization was carried out over 2 h at 68°C using the Quick Hyb^® ^solution. Post hybridization – washes were performed twice for 15 minutes at room temperature and twice for 5 – 15 minutes at 50°C in 2 × SSC containing 0.1% SDS. Nylon filters were exposed to X-ray films at -80° and finally densitometric scans were performed.

### Western Blot Analysis

Cells were lysed in hot Laemmli buffer (95°C) and processed by sodium dodecyl sulphate polyacrylamide gel electrophoresis (SDS-PAGE; 9% polyacrylamide) under reducing conditions [45]. The protein content of the cell lysates was calculated by the Coomassie Protein Assay (Pierce, Rockford, IL) and 30 μg protein per lane were applied. Proteins were transferred onto Hybond-ECL nitrocellulose membranes according to [46]. Immunodetection was performed according to the ECLWestern blotting protocol. Antibodies against p-STAT1 and p-SMAD2/3 were used at 2.5 μg/ml solutions, β-actin at 1:1000 solution, and peroxidase-labeled anti-mouse and anti-rabbit immunoglobulins were each used at a 1:1000 dilution.

### Statistical analysis

Densitometric analysis of the blots was performed using the program Scion Image Beta 4.0.2 (NIH, Washington, USA). Statistical evaluation was done by means of the Wilcoxon test.

## Abbreviations

APAAP: alkaline phosphatase-anti-alkaline phosphatase; CCl_4_: carbon tetrachloride; EC: endothelial cell; ICAM: intercellular adhesion molecule; IFN: interferon; Ig: immunoglobulin; FCS: foetal calf serum; MNP: Mononuclear phagocyte; PCR: polymerase chain reaction; PECAM: platelet endothelial cell adhesion molecule; SEC: sinusoidal endothelial cell; TGF: transforming growth factor; TNF: tumor necrosis factor.

## Authors' contributions

KN participated in the design and coordinated the study, performed and supervised the rat and mouse liver damage and drafted the manuscript. AL removed the tissues, isolated the cells and carried out the immunohistochemistry and FACS analysis. KT performed the molecular studies. GR participated in the design of the study and critically reviewed the manuscript. BS participated in the design and coordination of the study, performed the immunoblotting and ELISAs, interpreted the results, helped to draft the manuscript and critically reviewed and revised its final version. All authors have read and approved the manuscript.
